# The First National Remote Emergency System for Malignant Hyperthermia (MH-NRES) in China: Protocol for the Design, Development, and Evaluation of a WeChat Applet

**DOI:** 10.2196/37084

**Published:** 2022-06-10

**Authors:** Hong Yu, Lingcan Tan, Yi Teng, Zhao Xu, Kun Xiao, Jin Yin, Yunxia Zuo, Tao Zhu, Xiaoqian Deng

**Affiliations:** 1 Department of Anesthesiology Sichuan University West China Hospital Chengdu China; 2 School of Information and Software Engineering University of Electronic Science and Technology of China Chengdu China; 3 West China Biomedical Big Data Center Sichuan University West China Hospital Chengdu China

**Keywords:** malignant hyperthermia, hyperthermia, anesthetic, anesthesia, anesthesiology, anesthesiologist, mHealth, mobile health, health app, evaluation, user experience, perception, development, uni-app, digital health, national remote emergency system, WeChat, emergency, WeChat applet, dantrolene, China, Chinese, applet, messaging app, calling app, diagnosis, diagnostic service

## Abstract

**Background:**

Malignant hyperthermia (MH) is a rare life-threatening anesthetic emergency. With respect to the high fatality rate, difficulty in early recognition, and the lack of disease-specific drug (ie, dantrolene) in China, more effort is needed to strengthen early diagnosis and effective treatment of MH emergencies. Nowadays, mobile health (mHealth) apps are changing the way of medical practice; they can serve as an accessible tool to help anesthesiologists deal with MH crises. However, no related mHealth-based emergency system is available currently.

**Objective:**

The aim of this study is to outline the protocol for the development of a WeChat applet used to design a National Remote Emergency System for Malignant Hyperthermia (MH-NRES) in China, as well as the protocol for the evaluation of the user experience and perception of the system.

**Methods:**

The system adopts the client-server architecture, with a custom user interface operating as clients and the back-end system operating as the server. The client-side software was developed using uni-app technology with Vue.js-based framework, which consists of 6 modules: Quick Diagnosis, Dantrolene Mobilization, Instruction on Dantrolene Use, MH Treatment, Recovery Period Treatment, and DNA Test and Biopsy. The back-end system was developed based on the Spring framework. The system will be evaluated by administrating a modified user version of the Mobile App Rating Scale. Pilot testing will be conducted in Sichuan Province, China, and a subsequent evaluation on a national scale is planned.

**Results:**

The theoretical framework design of this system was completed in August 2021. The development of the system was completed in February 2022, and the refinement is currently ongoing. Pilot testing after the implementation of the system in Sichuan Province is planned to take 2 months, and the subsequent evaluation on a national scale is planned to take 2 months.

**Conclusions:**

We have described a novel approach using the WeChat applet to develop the MH-NRES. Findings from the usability testing process in the current study may lead to refinements and is expected to suggest that this system is both feasible and welcomed by anesthesiologists. Depending on the availability of research funding, this system will be extended nationally across China.

**International Registered Report Identifier (IRRID):**

PRR1-10.2196/37084

## Introduction

Malignant hyperthermia (MH) is a progressive life-threatening pharmacogenetic disorder of skeletal muscle occurring during general anesthesia. MH is usually triggered by exposure to any of the potent inhalational anesthetics or succinylcholine [1,2]. It is manifested by sustained skeletal muscle hypermetabolism related to altered calcium homeostasis [3]. MH is a rare anesthetic emergency. The incidence of MH reactions has been estimated to range from 1 per 10,000 to 1 per 150,000 general anesthetic procedures [4,5]. Nevertheless, MH should not be neglected as it is a fatal medical emergency in the operating room. However, the diagnosis of an acute MH reaction can be difficult because of the nonspecific nature and variable incidence of many of the clinical signs and laboratory findings [6]. Anesthesiologists need to recognize it in its early stages and begin appropriate management without any delay [7]. In China, there has been increased recognition and publication of MH cases in recent years [8-10]. Over the past 35 years, a reported total of 136 MH events occurred in mainland China according to data from the nonprofit academic organization China MH Emergency Assistance Group [11]. However, knowledge about the recognition and management of MH is still lacking among anesthesiologists [8]. The mortality rate of MH was as high as 55.9% [11], which was similar to the range reported in the pre-dantrolene era in the United States [2]. More effort is needed to strengthen early diagnosis and effective treatment of MH cases in the Chinese medical community, especially among anesthesiologists.

Furthermore, the lack of intravenous (IV) dantrolene in most Chinese hospitals is the major limitation in MH treatment [8]. Dantrolene is the only disease-specific drug available for MH. The fatality rate of MH has dropped from 70% in the 1970s to 9.5% after IV dantrolene became commercially available in the United State in 1981, according to a report from the Malignant Hyperthermia Association of the United States (MHAUS) [2]. Nevertheless, few hospitals in China stored IV dantrolene because the imported IV dantrolene was not on the list of government-approved drugs in China in the past. To address this problem, domestic IV dantrolene was produced by a Chinese pharmaceutical company (Livzon Pharmaceutical Group Co) and approved by the Chinese Food and Drug Administration in 2020. However, it is difficult for IV dantrolene to be stocked in all Chinese hospitals where general anesthesia is performed because of the low incidence and poor awareness of MH and the high cost of dantrolene. A more realistic solution would be that IV dantrolene is stored in a portion of the major hospitals within a reasonable distance and mobilized as soon as possible when an MH emergency occurs.

In recent years, as smartphones have become widely used in China, mobile health (mHealth) apps are changing the way of medical practice. Given the high fatality rate of MH and lack of information among anesthesiologists in China, it is necessary to implement a national remote emergency system for MH. However, no mHealth apps that can help anesthesiologists deal with MH crises were available until now. We planned to develop this system as an applet of the WeChat app. This would have several benefits. First, WeChat is an extremely popular social app in China [12]. By 2016, WeChat was installed in more than 94% of smartphones in China. When needed, anesthesiologists could find this system quickly by searching for this applet in WeChat without taking time to download a new app. Second, the applet in WeChat is easy to operate and can offer multiple functions, such as text and voice messages, free voice and video calls, group chats, and subscription to public accounts. Therefore, this WeChat applet might have the potential to help anesthesiologists improve the management of MH in emergency situations.

The primary aim of this paper is to outline the protocol for the development of a WeChat applet used to design the National Remote Emergency System for Malignant Hyperthermia (MH-NRES), a free mHealth WeChat applet providing a paperless, user-friendly solution for quick diagnosis, the rapid initiation of MH treatment, and dantrolene mobilization. The secondary aim of the paper is to describe the protocol for the evaluation of the user experience and perception of the system.

## Methods

### Software System Development

#### Basic Applet Conception

We built the MH-NRES as an applet of WeChat. We took into account several important considerations of the design. First, the applet should ensure quick access without the hindrances of registration and authorization (not exceeding >1 minute). Second, the system needs to be user friendly with ease for quick data entry (not exceeding >2 minutes). Third, the accuracy of all MH-related medical knowledge should be ensured. Fourth, there needs to be a back-end server that stores the data for future analysis. Finally, all information related to MH must be presented in a way that is easily read and interpreted.

#### Technical Specifications

The system adopts the client-server architecture, with a custom user interface (UI) operating as clients (service requesters) and the back-end system operating as the server (service provider) to securely store the data.

The client-side software was developed using uni-app technology. Uni-app is a Vue.js-based framework for software developers, with good cross-platform compatibility (ability to work on multiple operating systems; eg, it supports Android, iOS, H5, and multiple applet formats). The Vue.js-based framework features application programming interfaces, which only focuses on view layers. It simplifies the integration with third-party databases or existing projects. The interactive UI of the system uses ColorUI (version 2.1.6; Xiaogang Wen). ColorUI is a highly customizable Cascading Style Sheets styles gallery, providing common elements and components that can be integrated into other elements or components.

The back-end system was developed based on the Spring framework. Spring is an open-source app framework on the Java platform that includes containers with the inversion of control characteristics and provides a series of solutions for development. The app server follows the hierarchical structure of SpringBoot—Data Access Object, Service, and Controller. The Controller layer allows the client side to implement logic task by accessing the application programming interfaces. We used a widely used database management system called Oracle database to capture, query, and administer the data collected by the applet. As a general database system, it has complete data management functions.

### UI Design

#### Design Overview

**Figure 1:**
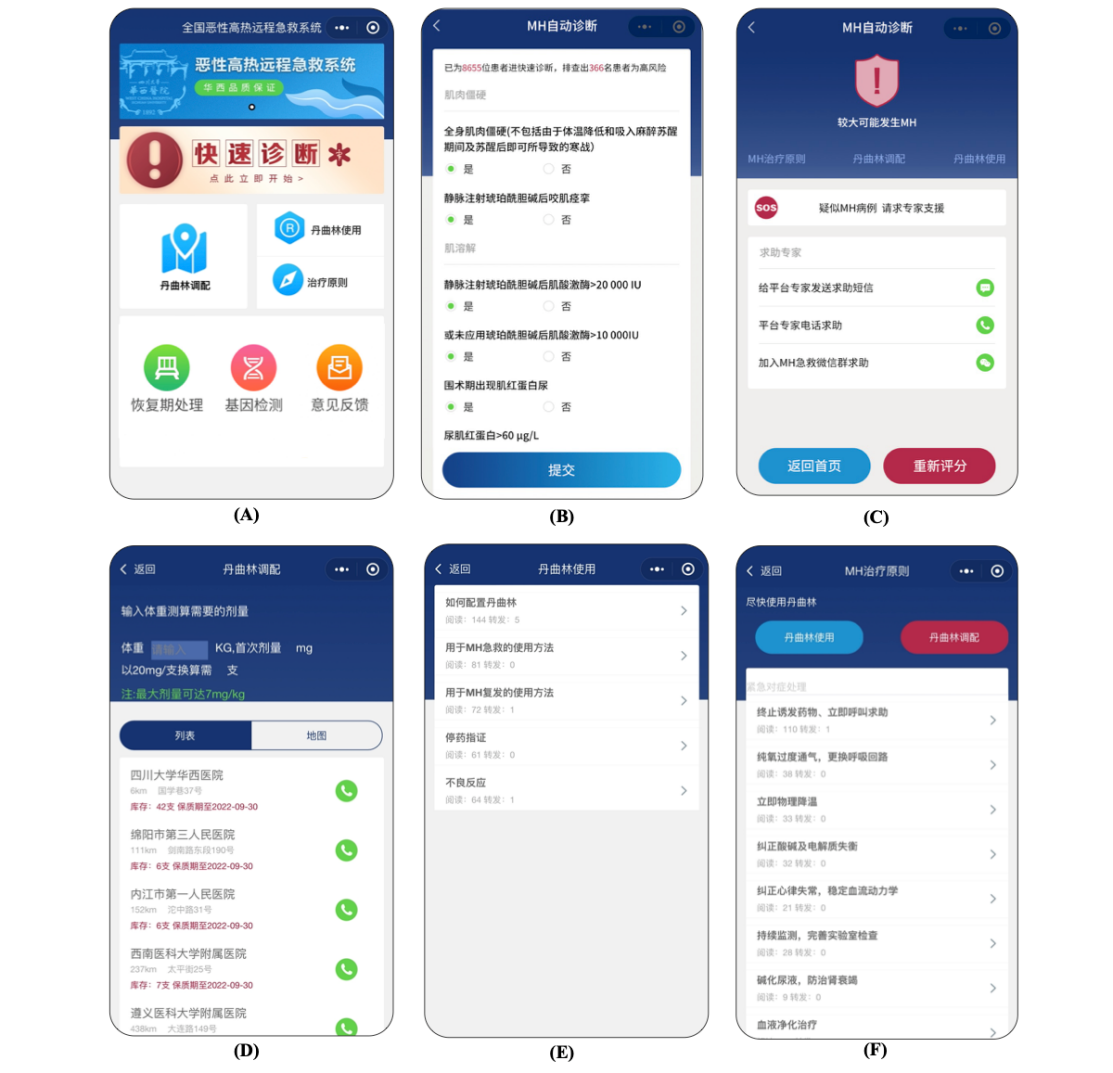
The user interface design of the National Remote Emergency System for Malignant Hyperthermia (MH-NRES), including (A) the Home Page, (B) the Quick Diagnosis forum, (C) an example of Malignant Hyperthermia (MH) rank and the corresponding recommendation in the Quick Diagnosis forum, (D) the Dantrolene Mobilization forum, (E) the Instruction on Dantrolene Use forum, and (F) the MH Treatment forum.

The theoretical framework design of UI used MindMaster Software (version 9.0.4; Yitu Software). The UI was designed to be user friendly with little-to-no training time. The content is written in Chinese. The UI provides forms with checkboxes, radio buttons, text-input boxes, and numeric sliders to make data input quick and simple. This system permits access to all anesthesiologists (and is not limited to anesthesiologists). When opening the applet, the user is asked to log in with their smartphone number and dynamic certification code and provide the purpose of this log-in (review or an encounter with a suspected MH patient). The back-end server will store this information which would facilitate the data tracking of suspected MH cases and subsequently the establishment of the database. There are 6 major modules visibly placed on the home page including Quick Diagnosis, Dantrolene Mobilization, Instruction on Dantrolene Use, MH Treatment, Recovery Period Treatment, and DNA Test and Biopsy (Figure 1A).

#### Quick Diagnosis

The Quick Diagnosis forum is the most prominent part on the home page, which can be quickly seen by users. Users can make a quick diagnosis by self-diagnosing according to the Scoring Rule for the MH Clinical Grading Scale [6,13] (Figure 1B). The Clinical Grading Scale provides the qualitative likelihood of an MH event, which consists of 7 categories: rigidity, muscle breakdown, respiratory acidosis, temperature increase, cardiac involvement, family history, and other indicators. Each category corresponds to several clinical indicators. Users can use the checkboxes to select each present clinical indicator, and all inputs are stored on the server and accessible to the user for re-evaluation. Points are assigned for each present clinical indicator—if more than 1 indicator represents a single category, only the indicator with the highest score is counted; these points are then added to produce a raw score. The raw score was designed to be translated into an MH rank designating the risk of MH occurring, from 1 (almost never) to 6 (almost certain; Multimedia Appendix 1). Different MH ranks corresponds to different intervention recommendations (an example of MH rank 5 and the corresponding recommendation is shown in Figure 1C). For MH ranks 1 and 2, the recommendation is “Re-evaluation” or “Differential Diagnosis”; for MH rank 3, the recommendation is “Re-evaluation” and to observe the patients closely; for MH ranks 4 to 6, a sparkling “SOS” will emerge to alert the anesthesiologists of a highly likely MH crisis, and “MH Treatment,” “Dantrolene Mobilization,” and “Dantrolene Use” are recommended. The texts of “Re-evaluation,” “Differential Diagnosis,” “MH Treatment,” “Dantrolene Mobilization,” and “Dantrolene Use” were designed as tabs hyperlinked to new UIs to give the users detailed information. After self-diagnosing, alternatively, anesthesiologists can ask for help from experts either through the experts’ hotline or by contacting the administrator (LT) to join the China MH Emergency Assistance WeChat Group.

#### Dantrolene Mobilization

If the likelihood of an MH event is graded at ranks 4-6, then dantrolene administration is the most important intervention. Users could use the function of Dantrolene Mobilization when dantrolene is not available in their hospital. In this forum, dantrolene could be mobilized in 3 steps (Figure 1D). Step 1 involves calculating the minimum number of dantrolene vials needed according to the formula. The minimum dose corresponds to the initial dose administrated (1 mg/kg for Asian people based on actual bodyweight) [13]. Step 2 involves finding the appropriate drug suppliers. A list of the nationwide drug suppliers (hospitals or pharmaceutical companies) with available preparations was established. The information of the suppliers’ name and location, the distance away from the user’s hospital, the amount of the drug in stock, and contact telephone number were collected and presented. We established the following rules to order the drug suppliers displayed in the UI: (1) the shortest distance away from the users’ hospital, and (2) the amount of the drug in stock exceeding the minimum initial dosage. According to the geographical location of the users and the quantity of dantrolene needed, the institutions that can provide dantrolene are intelligently selected and displayed in order. Step 3 involves selecting an institution and dialing the contact number to start dantrolene mobilization.

#### Instruction on Dantrolene Use

This forum provides information on how to use dantrolene. The instruction on dantrolene use is based on the Malignant hyperthermia 2020 Guideline from the Association of Anesthetists [7] and the drug direction of the domestic dantrolene sodium injection produced by Livzon Pharmaceutical Group Co. The UI consists of 5 parts: Preparation of Dantrolene, Dantrolene for Acute MH Reaction, Dantrolene for MH Recurrence, Treatment Goals, and Side Effects (Figure 1E). Each part is hyperlinked to the corresponding text description when clicked by the users [14] (Multimedia Appendix 2).

#### MH Treatment

According to the principles of treatment, this forum lists the management protocol of an MH emergency (eg, administer dantrolene, notify the surgeon, discontinue triggering agents, optimize oxygenation and ventilation, etc; Figure 1F). Each intervention is a tab hyperlinked to a new UI to give the users detailed information [7] (Multimedia Appendix 3).

#### Recovery Period Treatment

This forum consists of 3 parts: Monitoring, Clinical Signs for Recurrence, and Dantrolene for MH Recurrence. Each part is a tab hyperlinked to a new UI to give the users detailed information [7] (Multimedia Appendix 4).

#### DNA Test and Biopsy

This forum provides diagnostic services for suspected MH patients and their family. There are 2 options for the investigation of MH susceptibility [15]. The first option is DNA screening, which is relatively cheap, minimally invasive, and convenient for the patients. The “DNA Test” part provides users with the information of the available centers for DNA testing and instructions for blood sampling. Notably, DNA screening only has approximately 50% sensitivity for detecting MH susceptibility [16]. A definitive diagnosis of MH susceptibility relies on specialized tests carried out on freshly excised muscle strips taken from open biopsy (the in vitro contracture test) [15]. Unfortunately, the muscle biopsy test is not conducted in China, so the “Biopsy” part only provides users with some theoretical knowledge about biopsy.

### Evaluation of the MH-NRES

#### Modified User Version of the Mobile App Rating Scale

After the development of a fully functional smartphone-based prototype, usability testing of this system will be conducted with a modified user version of the Mobile App Rating Scale (uMARS) in 2 stages. The app interface designs will be trialed with anesthesiologists. The first stage is a pilot test that will be conducted by informal user reviews from 20 members of the Anesthesiology Committee of Sichuan Medical Association in Sichuan Province. The second stage is a nationwide sample survey. The target participants are 20 anesthesiologists working in the top 10 Chinese university–affiliated hospitals of anesthesiology. The uMARS is derived from the Mobile App Rating Scale and is a validated mobile app evaluation tool that has been used extensively to rate the quality of medical apps [17]. The original version of uMARS comprises the following 3 domains to evaluate an app using a Likert-type rating scale: (1) quality score, which examines engagement, functionality, aesthetics, and content information; (2) subjective quality, which questions the likelihood of recommending the app to others, use in the future, overall rating, etc; and (3) behavior change, which assesses the perceived impacts on knowledge, attitude, awareness, and behavior [17]. The modified uMARS comprises 25 questions by retaining those questions that are relevant to the current applet. Since the applet was designed to be used as an aid when an adverse anesthetic event (clinical MH) occurs, we excluded the items evaluating entertainment and interest in the engagement domain. As the applet is free and a nonprofit property, we excluded the question “Would you pay for this applet?” Moreover, user perceptions will be collected with 2 open-ended questions: (1) If you decide to use or not use this app, what are the possible reasons for it? and (2) What improvements do you want to see in future versions of the app? (Multimedia Appendix 5)

#### Data Analysis

SPSS statistical software (version 23.0; IBM Corp) will be used for all quantitative data analysis including participant demographic data and the subscales and total scores of the uMARS. Descriptive statistics will be used to show continuous variables as mean (SD) and categorical variables as frequency and percentage. The open-ended questions will be reviewed by 2 researchers (HY and LT) independently. Qualitative data will be organized into meaningful groups by combining similar patterns into themes.

### Quality Control

Quality control strategies are being implemented. The principal investigator (XD) works closely with research staff in the applet design, development, and evaluation process. Team meetings are conducted every 1 or 2 weeks for progress updates and problem-solving. Discussion and debriefing are conducted promptly if needed. All the knowledge about MH in this system is derived from the Malignant hyperthermia 2020 Guideline from the Association of Anesthetists [7] in China and the UpToDate website [18].

### Ethics Approval

The trial was conducted in accordance with the Declaration of Helsinki (as revised in 2013). This trial was approved by the Institutional Review Board of West China Hospital of Sichuan University on November 11, 2021 (Ethical number: 2021-1446). All data will be treated anonymously to protect personal privacy. Private information such as patients’ name and ID number will not be input during any operating steps. If the experts’ hotline is initiated, only the patient’s hospital will be disclosed. Informed consent will be sought from patients before subsequent gene diagnosis, family counseling, and MH database construction.

## Results

The theoretical framework design of this system was completed in August 2021. The development of the system was completed in February 2022, and the refinement is currently ongoing. Pilot testing after the implementation of the system in Sichuan Province is planned to take 2 months, and the subsequent survey on a national scale is planned to take 2 months. The project is supported by the National Natural Science Foundation of China (72074162).

## Discussion

### Comparison With Prior Work

MH is a rare life-threatening anesthetic emergency, which has been found to occur worldwide including in China. The current status of MH in mainland China is still not optimistic as problems regarding the underestimation of the incidence, poor recognition and diagnosis, and the lack of specific treatment still exist [19]. This study will provide valuable information on the development and evaluation of the MH-NRES, which is aimed at providing a real-time and effective emergency system to help Chinese anesthesiologists deal with MH crises.

In the United States, a nonprofit organization, MHAUS, provides a 24-hour MH hotline to give real-time advice to professionals in handling MH crises. However, this approach may not be suitable for Chinese users. First, the language barrier cannot be ignored. Second, a hotline is not as readily available in the operating room as smartphone apps. Third, it cannot support the dantrolene mobilization that is practical and necessary in China. Nevertheless, Chinese experts have made a lot of efforts to improve the management of MH. A WeChat chat group (China MH Emergency Assistance Group) was established in 2015. This chat group is composed of MH experts from large academic medical centers and anesthesiologists from different levels of hospitals in China. However, it has some limitations. First, this group can only accommodate 500 members from 300 hospitals with many grassroots hospitals excluded. Second, the system highly relies on real-time direction from experts, but the help-seeking message might be obscured by other irrelevant messages. Third, anesthesiologists need to filter the suitable information from a large number of expert suggestions, which may be time-consuming and beyond their ability. Compared to the MHAUS and China MH Emergency Assistance Group, the MH-NRES might be a more effective real-time emergency system for MH in China. The MH-NRES is available for free and can be accessed quickly by all anesthesiologists. The system provides standard and evidence-based knowledge of MH and updated, real-world information of the amount of dantrolene in stock. The use of this system can assist anesthesiologists in China to make rapid diagnosis, implement effective management, initiate dantrolene mobilization in real time when MH cases occur, and provide subsequent gene diagnostic services and family counseling. The results from the user evaluation in this study will improve future versions of the system. The long-term impact of this system is expected to be beneficial, as it could increase anesthesiologists’ ability to deal with MH crises, increase dantrolene use, and consequently improve the prognosis of MH patients.

### Strengths and Limitations

The WeChat applet–based MH-NRES is different from other mHealth-based emergency system of MH (ie, the WeChat help groups) in China. First, besides providing direction to the experts’ hotline, this system can guide anesthesiologists to conduct self-diagnosis and manage MH. Second, it displays all service types without requiring specific user identification, and there is no limitation to the number of people online. Third, this study provides a guide to the architecture and framework for developing a national remote emergency system, which can be applied to design similar systems for other clinical emergencies. Fourth, the data collected by the system are helpful to building the first national MH database and obtaining a better estimate of the incidence of MH in China. With respect to the limitations, we expect that the results from the evaluation of user experience and perception could help us improve future versions of the system.

### Conclusions

We have described a novel approach using the WeChat applet to develop the MH-NRES. Findings from the usability testing process in this study may lead to refinements and is expected to suggest that this system is both feasible and welcomed by anesthesiologists. Depending on the availability of research funding, this system will be extended nationally across China.

## Appendix

Multimedia Appendix 1 Clinical indicators for use in determining the Malignant Hyperthermia (MH) raw score and the corresponding MH rank.

Multimedia Appendix 2 Mapping of the Instruction on Dantrolene Use forum.

Multimedia Appendix 3 Mapping of the Malignant Hyperthermia Treatment forum.

Multimedia Appendix 4 Mapping of the Recovery Period Treatment forum.

Multimedia Appendix 5 Modified user version of the Mobile Application Rating Scale (uMARS).

## Abbreviations

IV: intravenous
MH: malignant hyperthermia
MH-NRES: National Remote Emergency System for Malignant Hyperthermia
MHAUS: Malignant Hyperthermia Association of the United States
mHealth: mobile health
UI: user interface
uMARS: user version of the Mobile App Rating Scale
