# Supplementary material for: The First National Remote Emergency System for Malignant Hyperthermia (MH-NRES) in China: Protocol for the Design, Development, and Evaluation of a WeChat Applet
Source: JMIR Res Protoc. 2022 Jun 10;11(6):e37084. doi: 10.2196/37084 (PMC9233253; doi:10.2196/37084)
Supplement: Multimedia Appendix 1 [file resprot_v11i6e37084_app1.docx]

**Multimedia Appendix1** Clinical indicators for use in determining the MH raw score and the corresponding MH rank.

| **Process** | **Indicator** | **Point** |
| --- | --- | --- |
| Process I: Rigidity | Generalized muscular rigidity (in absence of shivering due to hypothermia, or during or immediately following emergence from inhalational general anesthesia) | 15 |
|  | Masseter spasm shortly following succinylcholine administration | 15 |
| Process Il: Muscle Breakdown | Elevated creatine kinase >20,000 IU after anesthetic that included succinylcholine | 15 |
|  | Elevated creatine kinase>10,000 IU after anesthetic without succinylcholine | 15 |
|  | Cola colored urine in perioperative period | 10 |
|  | Myoglobin in urine >60 μg/L | 5 |
|  | Myoglobin in serum >170 μg/L | 5 |
|  | Blood/plasma/serum K^+^>6 mmol/L (in absence of renal failure) | 3 |
| Process Ill: Respiratory Acidosis | PETco_2_>55 mmHg with appropriately controlled ventilation | 15 |
|  | Arterial Paco_2_>60 mmHg with appropriately controlled ventilation | 15 |
|  | PETco_2_>60 mmHg with spontaneous ventilation | 15 |
|  | Arterial Paco_2_>65 mmHg with spontaneous ventilation | 15 |
|  | Inappropriate hypercarbia (in anesthesiologist's judgment) | 15 |
|  | Inappropriate tachypnea | 10 |
| Process IV: Temperature Increase | Inappropriately rapid increase in temperature (in anesthesiologist's judgment) | 15 |
|  | Inappropriately Increased temperature >38.8℃ in the perioperative period (in anesthesiologist's judgment) | 10 |
| Process V: Cardiac Involvement | Inappropriate sinus tachycardia | 3 |
|  | Ventricular tachycardia or ventricular fibrillation | 3 |
| Process VI: Family History (used to determine MH susceptibility only) | Positive MH family history in relative of first degree | 15 |
|  | Positive MH family history in relative not of first degree | 5 |
| Other indicators that are not part of a single process (These should be added without regard to double-counting) | Arterial base excess more negative than-8 mmol/L | 10 |
|  | Arterial pH <7.25 | 10 |
|  | Rapid reversal of MH signs of metabolic and/or respiratory acidosis with iv dantrolene | 5 |
|  | Positive MH family history together with another indicator from the patient's own anesthetic experience other than elevated resting serum creatine kinase | 10 |
|  | Resting elevated serum creatine kinase* (in patient with a family history of MH) | 10 |

| **Raw Score Range** | **MH Rank** | **Description of Likelihood** |
| --- | --- | --- |
| 0 | 1 | Almost never |
| 3-9 | 2 | Unlikely |
| 10-19 | 3 | Somewhat less than likely |
| 20-34 | 4 | Somewhat greater than likely |
| 35-49 | 5 | Very likely |
| ≥50 | 6 | Almost certain |
