# Supplementary material for: The First National Remote Emergency System for Malignant Hyperthermia (MH-NRES) in China: Protocol for the Design, Development, and Evaluation of a WeChat Applet
Source: JMIR Res Protoc. 2022 Jun 10;11(6):e37084. doi: 10.2196/37084 (PMC9233253; doi:10.2196/37084)
Supplement: Multimedia Appendix 2 [file resprot_v11i6e37084_app2.docx]

**Appendix 2** Mapping of Instruction on Dantrolene Use forum

***Preparation of dantrolene:*** The domestic dantrolene formulation is presented in 20 mg vials and each vial is reconstituted with 60 ml of sterile water with 5 min of vigorous shaking. Dantrolene should not be soluble in 5% glucose, 0.9% sodium chloride and any other solvents.

***Dantrolene for acute MH reaction:*** We recommend the initial 1 mg/kg based on actual bodyweight with further boluses of 1 mg/kg administered until the treatment goals are achieved or when a cumulative dose of 7 mg/kg dantrolene has been administered.

***Dantrolene for MH recurrence:*** If there is a rebound increase in EtCO_2_ and temperature, further doses are required.

We recommend the initial 1 mg/kg based on actual bodyweight, with further infusion of 0.25mg/kg/h administered at least 24h or further boluses of 1mg/kg every 4-6 hours until the treatment goals are achieved.

***Treatment goals:*** The recommended treatment goals are as follows:

- reduction of EtCO_2_ to less than 45mmHg with normal minute ventilation
- a core temperature < 38.5°C
- no muscle rigidity
- no cola-colored urine
- sustaining decreased creatine kinase

When these goals have been achieved, the administration of dantrolene should be paused.

***Side effects:*** Its most common local adverse reaction is venous irritation or thrombosis at the site of administration due to its high pH; side effects include nausea, malaise, lightheadedness, and mild to moderate muscle weakness. Respiratory muscle weakness may occur when larger doses are used, especially in patients who are debilitated
