# Supplementary material for: The First National Remote Emergency System for Malignant Hyperthermia (MH-NRES) in China: Protocol for the Design, Development, and Evaluation of a WeChat Applet
Source: JMIR Res Protoc. 2022 Jun 10;11(6):e37084. doi: 10.2196/37084 (PMC9233253; doi:10.2196/37084)
Supplement: Multimedia Appendix 3 [file resprot_v11i6e37084_app3.docx]

**Multimedia Appendix 3** Mapping of MH Treatment forum

***Administer dantrolene:*** as soon as the drug is reconstituted. (Dantrolene mobilization and Instruction in Dantrolene use are designed as tab here to be hyperlinked to the corresponding forums)

***Notify the surgeon:*** Halt the surgical procedure as soon as possible.

***Discontinue triggering agents:*** Immediately discontinue volatile anesthetic agents and increase fresh gas flow to ≥10 L/minute to enhance elimination of anesthetic gas. If available, insert charcoal filters into the inspiratory and expiratory limbs of the anesthesia breathing circuit, after flushing the circuit for ≥90 seconds with fresh gas. It is not necessary to change the anesthesia machine. If surgery must be continued, maintain general anesthesia with intravenous non-triggering agents, typically propofol.

***Optimize oxygenation and ventilation:*** Increase inspired oxygen to 100%. Increase ventilation rate and/or tidal volume to maximize ventilation and reduce the EtCO_2_. If the patient is not intubated, an endotracheal tube should be placed, using only non-depolarizing muscle relaxants if paralysis is required.

***Commence active body cooling:*** Institute cooling for patients with core temperature >39°C, and discontinue cooling when temperature decreases to 38°C. External cooling with a circulating water mattress or ice packs should be instituted first, followed by infusion of refrigerated IV fluid (20 mL/kg IV) as necessary. If further cooling is necessary, consider cold saline lavage of open body cavities.

***Blood gas monitoring and treatment of abnormalities*:** Measure electrolytes, blood gases for acid/base status.

- Hyperkalemia: Treat hyperkalemia (ie, calcium, bicarbonate, and insulin-glucose) to prevent the development of life-threatening arrhythmias or cardiac arrest. Treat hyperkalemia in patients with abnormal ECG waveforms (eg, peaked T waves, ventricular arrhythmias), or patients with potassium of ≥6 mmol/L even in the absence of ECG abnormalities.
- Metabolic acidosis: Consider bicarbonate (1-2 mmol/kg intravenous [IV], maximum dose 50 mmol) for base deficit greater than 8 mmol/L.

***Cardiovascular support:***

- Treat cardiac arrhythmias. Dysrhythmias usually respond to the treatment of acidosis and hyperkalemia.
- Avoid calcium channel blockers: Use of calcium channel blockers to treat hypertension during an acute MH crisis is contraindicated because of the possibility that it can worsen hyperkalemia and hypotension when co-administered with dantrolene; however, dantrolene should never be withheld as treatment for MH in patients receiving preoperative maintenance therapy with calcium channel blockers.

***Prevention of acute kidney injury (AKI):***

- Monitor urine output: Insert a bladder catheter to monitor urine color and volume and myoglobinuria
- Maintain urine output at 1 to 2 mL/kg/hour
- Alkalinization of the urine by administration of sodium bicarbonate
- Renal replacement therapy as necssary

***Prevention of disseminated intravascular coagulopathy (DIC):*** We recommend empirical treatment using platelets, fresh frozen plasma and cryoprecipitate. Tranexamic acid is not indicated in this situation.
