# Supplementary material for: The First National Remote Emergency System for Malignant Hyperthermia (MH-NRES) in China: Protocol for the Design, Development, and Evaluation of a WeChat Applet
Source: JMIR Res Protoc. 2022 Jun 10;11(6):e37084. doi: 10.2196/37084 (PMC9233253; doi:10.2196/37084)
Supplement: Multimedia Appendix 4 [file resprot_v11i6e37084_app4.docx]

**Multimedia Appendix 4** Mapping of Recovery Period Treatment forum

***Monitoring:*** Measure EtCO_2_, temperature, electrolytes, blood gases for acid/base status, creatine kinase (CK), and depending on the patient’s condition, serum or urine myoglobin, and coagulation parameters.

***Clinical sign for recurrence:***

- Elevated EtCO_2_ with respiratory acidosis
- Inappropriately rapid increase in temperature
- Inappropriately muscle rigidity
- Inappropriately metabolic acidosis

***Dantrolene for MH recurrence:*** If there is a rebound increase in EtCO_2_ and temperature, further doses are required. We recommend the initial 1 mg/kg based on actual bodyweight, with further infusion of 0.25mg/kg/h administered at least 24h or further boluses of 1mg/kg every 4-6 hours until the treatment goals are achieved.
