# Supplementary material for: The First National Remote Emergency System for Malignant Hyperthermia (MH-NRES) in China: Protocol for the Design, Development, and Evaluation of a WeChat Applet
Source: JMIR Res Protoc. 2022 Jun 10;11(6):e37084. doi: 10.2196/37084 (PMC9233253; doi:10.2196/37084)
Supplement: Multimedia Appendix 5 [file resprot_v11i6e37084_app5.docx]

**Multimedia Appendix 5** Modified user-version of Mobile Application Rating Scale (uMARS)

All items are rated on a 5-point scale from “1.Inadequate” to “5.Excellent”.

**Applet Quality Ratings**

**A．Engagement** –customizable, interactive (e.g., sends alerts, messages, reminders, feedback, enables sharing), well-targeted to audience

**Q1.** Customization: Does it provide/retain all necessary settings/preferences for applet features (e.g., sound, content, notifications, etc.)?

1 Does not allow any customization or requires setting to be input every time

2 Allows insufficient customization limiting functions

3 Allows basic customization to function adequately

4 Allows numerous options for customization

5 Allows complete tailoring to the individual’s characteristics/preferences, retains all settings

**Q2.** Interactivity: Does it allow user input, provide feedback, contain prompts (reminders, sharing options, notifications, etc.)? Note: these functions need to be customizable and not overwhelming in order to be perfect.

1 No interactive features and/or no response to user interaction

2 Some, but not enough interactive features which limits applet’s functions

3 Basic interactive features to function adequately

4 Offers a variety of interactive features/feedback/user input options

5 Very high level of responsiveness through interactive features/feedback/user input options

**Q3.** Target group: Is the applet content (visual information, language, design) appropriate for your target audience?

1 Completely inappropriate/unclear/confusing

2 Mostly inappropriate/unclear/confusing

3 Acceptable but not targeted. May be inappropriate/unclear/confusing

4 Well-targeted, with negligible issues

5 Perfectly targeted, no issues found

**A. Engagement mean score =**

**B. Functionality**–applet functioning, easy to learn, navigation, flow logic, and gestural design of applet

**Q4.** Performance: How accurately/fast do the applet features (functions) and components (buttons/menus) work?

1 Applet is broken; no/insufficient/inaccurate response (e.g., crashes/bugs/broken features, etc.)

2 Some functions work, but lagging or contains major technical problems

3 Applet works overall. Some technical problems need fixing/Slow at times

4 Mostly functional with minor/negligible problems

5 Perfect/timely response; no technical bugs found/contains a ‘loading time left’ indicator

**Q5.** Ease of use: How easy is it to learn how to use the applet; how clear are the menu labels/icons and instructions?

1 No/limited instructions; menu labels/icons are confusing; complicated

2 Useable after a lot of time/effort

3 Useable after some time/effort

4 Easy to learn how to use the applet (or has clear instructions)

5 Able to use applet immediately; intuitive; simple

**Q6.** Navigation: Is moving between screens logical/accurate/appropriate/ uninterrupted; are all necessary screen links present?

1Different sections within the applet seem logically disconnected and random/confusing/navigation is difficult

2 Usable after a lot of time/effort

3 Usable after some time/effort

4 Easy to use or missing a negligible link

5 Perfectly logical, easy, clear and intuitive screen flow throughout, or offers shortcuts

**Q7.** Gestural design: Are interactions (taps/swipes/pinches/scrolls) consistent and intuitive across all components/screens?

1 Completely inconsistent/confusing

2 Often inconsistent/confusing

3 OK with some inconsistencies/confusing elements

4 Mostly consistent/intuitive with negligible problems

5 Perfectly consistent and intuitive

**B. Functionality mean score = ____________**

**C. Aesthetics**–graphic design, overall visual appeal, color scheme, and stylistic consistency

**Q8.** Layout: Is arrangement and size of buttons/icons/menus/content on the screen appropriate or zoomable if needed?

1 Very bad design, cluttered, some options impossible to select/locate/see/read device display not optimized

2 Bad design, random, unclear, some options difficult to select/locate/see/read

3 Satisfactory, few problems with selecting/locating/seeing/reading items or with minor screen-size problems

4 Mostly clear, able to select/locate/see/read items

5 Professional, simple, clear, orderly, logically organized, device display optimized. Every design component has a purpose

**Q9.** Graphics: How high is the quality/resolution of graphics used for buttons/icons/menus/content?

1 Graphics appear amateur, very poor visual design - disproportionate, completely stylistically inconsistent

2 Low quality/low resolution graphics; low quality visual design – disproportionate, stylistically inconsistent

3 Moderate quality graphics and visual design (generally consistent in style)

4 High quality/resolution graphics and visual design–mostly proportionate, stylistically consistent

5 Very high quality/resolution graphics and visual design-proportionate, stylistically consistent throughout

**Q10.** Visual appeal: How good does the applet look?

1 No visual appeal, unpleasant to look at, poorly designed, clashing/mismatched colors

2 Little visual appeal – poorly designed, bad use of color, visually boring

3 Some visual appeal – average, neither pleasant, nor unpleasant

4 High level of visual appeal – seamless graphics – consistent and professionally designed

5 As above + very attractive, memorable, stands out; use of color enhances applet features/menus

**C. Aesthetics mean score =** ______________

**D. Information**–Contains high quality information (e.g. text, feedback, measures, references) from a credible source

**Q11.**Quality of information: Is applet content correct, well written, and relevant to the goal/topic of the applet?

1 Irrelevant/inappropriate/incoherent/incorrect

2 Poor. Barely relevant/appropriate/coherent/may be incorrect

3 Moderately relevant/appropriate/coherent/and appears correct

4 Relevant/appropriate/coherent/correct

5 Highly relevant, appropriate, coherent, and correct

**Q12.** Quantity of information: Is the information within the applet comprehensive but concise?

1 Minimal or overwhelming

2 Insufficient or possibly overwhelming

3 OK but not comprehensive or concise

4 Offers a broad range of information, has some gaps or unnecessary detail; or has no links to more information and resources

5 Comprehensive and concise; contains links to more information and resources

**Q13.** Visual information: Is visual explanation of concepts-through charts/graphs/images/videos, etc. - clear, logical, correct?

1 Completely unclear/confusing/wrong or necessary but missing

2 Mostly unclear/confusing/wrong

3 OK but often unclear/confusing/wrong

4 Mostly clear/logical/correct with negligible issues

5 Perfectly clear/logical/correct

**Q14.** Credibility of source: does the information within the applet seem to come from a credible source?

1 Suspicious source

2 Lacks credibility

3 Not suspicious but legitimacy of source is unclear

4 Possibly comes from a legitimate source

5 Definitely comes from a legitimate/specialized source

**D. Information mean score =** ______________

**Applet Subjective quality**

**Q15.** Would you recommend this applet to people who might benefit from it?

1 I would not recommend this applet to anyone

2 There are very few people I would recommend this applet to

3 There are several people I would recommend this applet to

4 There are many people I would recommend this applet to

5 I would recommend this applet to everyone

**Q16.** How likely do you think you would use this applet if it was relevant to you?

1 Not at all

2 Between 1 and 3

3 Maybe

4 Between 3 and 5

5 Definitely

**Q17.** What is your overall(star) rating of the applet?

1 🟊 One of the worst applets I’ve used

2 🟊🟊

3 🟊🟊🟊 Average

4 🟊🟊🟊🟊

5 🟊🟊🟊🟊🟊 One of the best applets I've used

**Perceived impact**

**Q18.** Awareness–This applet has increased my awareness of the importance of MH

Strongly disagree Strongly Agree

1 2 3 4 5

**Q19.** Knowledge – This applet has increased my knowledge/understanding of the MH

Strongly disagree Strongly Agree

1 2 3 4 5

**Q20.** Attitudes – The applet has changed my attitudes toward improving the management of MH

Strongly disagree Strongly Agree

1 2 3 4 5

**Q21.** Intention to change – The applet has increased my intentions/motivation to improve the management of MH

Strongly disagree Strongly Agree

1 2 3 4 5

**Q22.** Help seeking – This applet would encourage me to seek further help to improve the management of MH (if I needed it)

Strongly disagree Strongly Agree

1 2 3 4 5

**Q23.** Behavior change – Use of this applet will help me to improve the management of MH

Strongly disagree Strongly Agree

1 2 3 4 5

**User Perception**

**Q24.** If you decide or decided not to use this applet, what will be the possible reasons for it?

**Q25.** What improvements do you want to see in the future versions of the applet?
